# Prospective marriage and divorce data on Norwegian cohorts of two-sex marriages from 1886 until 2018

**DOI:** 10.1016/j.dib.2022.108083

**Published:** 2022-03-21

**Authors:** Rune Zahl-Olsen

**Affiliations:** Department of child and adolescent mental health, Sørlandet Hospital, Kristiansand, Norway

**Keywords:** Marriage, Divorce, Remarriage, Prospective, Follow up, Urban, Rural

## Abstract

The article contains prospective data on all two-sex marriages formed in Norway between 1886 and 2018, with annual follow-up for 60 years, totaling 133 cohorts and 2 698 632 marriages. The data list the number of marriages that ended in divorce throughout each year of follow-up. The data were obtained through a special order from Statistics Norway. The data from 1981 to 2018 contain additional information about male and female ages, the number of divorces from each cohort in the total population of marriages, as well as divorces among marriages formed in urban and rural areas of the country under four marriage and remarriage constellations: first time for both spouses, first time for one and second time for the other, second time for both, and third time or more for at least one in the couple. Marriages formed within a calendar year are pooled into cohorts, and each pair was examined annually to ensure that the same two persons remained married.

## Specifications Table

**Table:** 

Subject	Social Sciences
Specific subject area	Marriage, remarriage and divorce
Type of data	Table
How data were acquired	The data on cohorts 1886 until 1980 stems from a report from Statistics Norway, published in Mamelund et al. (1987). Data on cohorts from 1981 to 2018 were obtained from Statistics Norway through a special request.
Data format	Raw
Parameters for data collection	All marriages formed in Norway from the beginning of 1886 until the end of 2018 were included.
Description of data collection	*For the 1886 -1980 dataset:* Three researchers at Statistics Norway reconstructed unpublished tables. From 1964 every citizen in Norway got a personal identification number, something that made it possible to follow individuals from one year to another. *For the 1981- 2018 dataset:* The dataset was created by one statistician at Statistics Norway who had access to data on every person and their marriage status. Each case was investigated more completely than a previous investigation (Zahl-Olsen and Shahar, 2021), resulting in a revised definition of included marriages as detailed in the Data Description section.
Data source location	Institution: Statistics Norway Country: Norway The major data source comprises information about every individual in Norway on a variety of characteristics, and hence contains sensitive information. Statistics Norway makes primary data available only to certified employers. However, researchers connected with a research institution certified by The Research Council of Norway or Eurostat may obtain microdata on a case-by-case basis.
Data accessibility	Repository name: Mendeley Data Data identification number: 10.17632/jx73wpcx2c.1 Direct URL to data: https://data.mendeley.com/datasets/jx73wpcx2c/draft?a=1bb2bb43-8014-444b-9895-87bd879f4a8b
Related research article	Zahl-Olsen, R. (2022). UNDERSTANDING DIVORCE TRENDS AND RISKS: THE CASE OF NORWAY 1886–2018. *Journal of Family History*[3].

## Value of the Data

• Many people suffer divorce, and earlier research has indicated an increased risk of divorce among those who remarry and those who marry in urban areas. However, urban and rural remarriages differ in several ways, and this dataset includes information on first marriages as well as three distinct forms of remarriage.
• This data may be of value to social scientists in Norway and other countries who are conducting research on committed partnerships and divorce trends.
• The data are of considerable practical importance since it may be used for making cross-cultural comparisons for first and different constellations of remarriages as well as for marriages formed in rural and urban areas. The data may be used to examine how patterns of relationship breakup have changed over time among European couples. When dissolution rates for distinct cohorts are compared at various time points during follow-up, tendencies emerge.

## 1 Data Description

The dataset [6] contains a 133-year time series of marriage and divorce statistics. Norwegian legislation governing adult relationships has evolved significantly over the time period. Divorce became legal in 1909. Cohabitation became legal in 1972. In 1993, a new divorce law made divorce easier and, in some situations, faster. Same-sex weddings became allowed in 2009. However, this data does not cover same-sex marriages. The marriage population has fluctuated throughout time. The data is organized in two Excel files with multiple sheets. Additionally, the data are provided as separate csv files.

*The first file, “Data Two sex marriages 1886 until 2018 – Total sample” has six sheets.*

**Graph 1:**
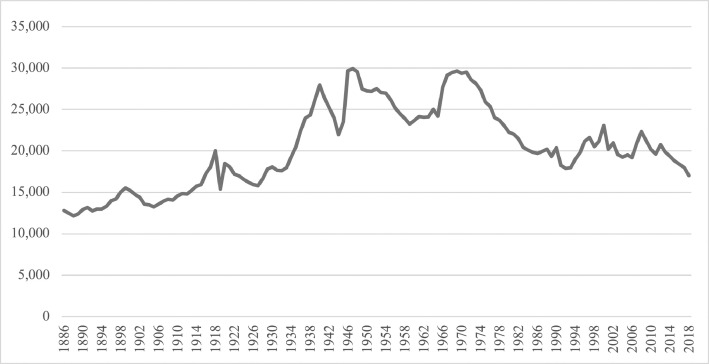
Number of marriages for each cohort from 1886 until 2018.

1. The first sheet, titled **Divorce rate all cohorts**, present aggregated divorce rate for each year of follow-up for the total population. In addition, it has a line for total divorce rate. That is the aggregated divorce rate in 2018. The data on this line correspond to the most recent follow-up data for each cohort, indicating that young and old cohorts have different years of follow-up. From 1886 to 1959, the cohorts received 60 years of follow-up, but subsequent cohorts received less years. Within each cohort, the population line indicates the size of the married population, or the number of weddings. The number of weddings each year has varied during the 133 years as illustrated in Graph 1. In comparison, Norway's population has grown gradually from 1.95 million in 1886 to 5.30 million in 2018.

**Graph 2:**
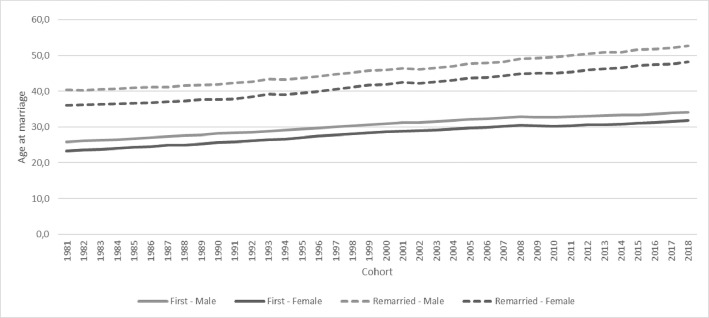
Age at marriage for males and females for each cohort, first marriages and remarriage.

2-5. Sheet two to five present four categories of marriages used in other studies [2,4] as described in the following:

• **First – first**, comprising all marriages where both spouses were married for their first time.
• **First – second**, comprising all marriages where one of the spouses had been married once before.
• **Second - second**, comprising all marriages where both spouses had been married once before.
• **Third or more**, comprising all marriages where at least one of the spouses had been married twice or more before.

6. The final sheet, labelled Age, specifies the present age at marriage with SD for the male and female partners in each cohort, for each of the four categories, and for remarriages combined, as illustrated in Graph 2.

The first five sheets present the data in the same way. Data for each cohort are presented in separate columns. The first line presents the cohort, e.g., 1981. The second line present the total population of marriages in that category of marriage (e.g., First – First) for each cohort. The fourth to the last line present the accumulative percentage of divorced from that cohort by the end of each year of follow-up (0 – 60 years from the year of marriage).

*The second file, “Data Two sex marriages 1981 until 2018 – Urban and Rural sample”,* contains data on marriages formed in Norway's urban and rural areas. Marriages were classified into two categories according to the population of the municipality in which the groom was registered at the time of the wedding. Norway had 356 municipalities in 2020. These municipalities were ordered from largest to lowest in terms of population. The urban sample included marriages from the six largest municipalities, including the five largest cities. The rural sample eliminated marriages from 27 municipalities with a medium population size and included marriages from the remaining 323 small municipalities. All municipalities included in the urban group had a population greater than 110 000, whereas all municipalities included in the rural group had a population less than 31 000. This strategy produced two groups of comparable size and targeted a significant cultural gap between the two groups. The six municipalities defined as urban included: Oslo, Bergen, Trondheim, Stavanger, Bærum, and Kristiansand. The 27 municipalities that were excluded were: Drammen, Asker, Lillestrøm, Fredrikstad, Sandnes, Tromsø, Ålesund, Sandefjord, Nordre Follo, Sarpsborg, Tønsberg, Skien, Bodø, Moss, Larvik, Arendal, Indre Østfold, Karmøy, Lørenskog, Ullensaker, Øygarden, Haugesund, Porsgrunn, Ringsaker, Molde, Halden and Hamar.

The data in this second file is structured differently than in the first. Each of the three sheets is presented in long format, with the following columns separating the subsamples:

• Category: Total, first-first, first-second, second-second, third or more. The definitions of each of these are the same as described for the first file.
• Region: Urban, Rural.
• Cohort: 1981 until 2018.

*The first* sheet, titled **Divorces**, holds the number of divorces for each year of follow-up for each cohort, region and category. It has the following two columns:

**Year divorced:** 1981 until 2018

**N divorced:** number of divorced during that year of follow-up for that cohort. The numbers for each subsequent year must be aggregated to determine the divorce rate within a cohort at a specified follow-up period, such as five years.

*The second* sheet, titled **Population**, contains the population for each cohort, region and category.

*The third* sheet, titled **Age**, contains the age and standard deviation of the male and female partners in each cohort, location, and category.

## 2 Experimental Design, Materials and Methods

Mamelund et al. [1] provided the data for the cohorts from 1886 to 1980. Since 1964, each person living in Norway has been assigned a unique identification number in Statistics Norway's database. If a person marries, this information is associated with their unique id. If the individual undergoes divorce, this will also be recorded and associated with that number. We used this data on individuals to track married couples to ensure that they were not only married for each succeeding year, but also married to the same person each year. This strategy ensured that we were able to trace each marriage and identify any divorces, even if a person divorced and remarried within the same year. Mamelund et al. [1] used different historical statistics for marriages formed prior to 1964, as mentioned in their report. For cohorts older than 1981, we included only marriages in which the husband resided in Norway. Newer cohorts include marriages in which one or both partners reside in Norway. For certain older cohorts (<1981), data are missing for each year of follow-up and are only available as aggregated data for three or five years. While some argue that when examining divorces, it is preferable to use reports of separation rather than divorce [5], however, this is a status both reversible and not always documented in the Norwegian database. In Norway, married couples seeking divorce usually first separate. Separated people are counted as married in this statistics because it is a situation they can withdraw from.

For many reasons, the statistics from 1981 to 2018 do not correspond to the publicly accessible data at ssb.no. To begin, each marriage is recorded in this study according to the year of marriage, not the year of registration, as is done in the official register. Second, during the data preparation process, we discovered that thousands of marriages were duplicated in the Statistics Norway database. As a result, we eliminated all probable duplicates (same two persons married again same year, or the year after, or where one was registered with a personal number and the other not in one registration and both in the other). Additionally, we eliminated marriages in which one spouse was unknown (e.g., that a spouse from another country was not registered with personal data and we could not follow-up to determine if they still were married in the coming years). This also distinguishes the data from Zahl-Olsen and Shahar's [2] study, which also examines marriage and divorce among cohorts from 1981 to 2013.

## Ethics Statement

Participant data are fully anonymized and are provided in compliance to data redistribution policies from Statistics Norway.

## CRediT authorship contribution statement

**Rune Zahl-Olsen:** Conceptualization, Methodology, Writing – original draft, Writing – review & editing.

## Declaration of Competing Interest

The author declares that he has no known competing financial interests or personal relationships that have or could be perceived to have influenced the work reported in this article.
